# Robotic pyelolithotomy in a congenital pelvic kidney: side docking and robotic prostatectomy port - site approach

**DOI:** 10.1590/S1677-5538.IBJU.2016.0059

**Published:** 2017

**Authors:** Rawan Al-Yousef, Ahmad Almarzouq, Saad Aldousari

**Affiliations:** 1 Faculty of Medicine, Kuwait University, Kwait;; 2 Urology Unit, Department of Surgery, Mubarak Alkabir Hospital, Kuwait;; 3 Urology Unit, Department of Surgery, Faculty of Medicine, Kuwait University, Kwait

## Abstract

**Introduction and Objectives:**

Ectopic pelvic kidneys with renal stones are challenging to treat. We report our experience in managing a case of ectopic pelvic kidney with a pelvic stone by robotic pyelolithotomy after failure of flexible ureteroscopy.

**Materials and Methods:**

A 46-year old male with 2 months history of vague lower abdominal pain was found to have on Computed Tomography scan a left ectopic pelvic kidney with a 12mm stone in an anomalous renal pelvis. Flexible ureteroscopy failed to reach the stone twice and a 4.7 French ureteric stent was placed.

**Results:**

Side docking was utilized with the patient in supine Trendelenburg position. Port placements were similar to robotic assisted laparoscopic prostatectomy. Docking time was 35 minutes and console time was 150 minutes. Multiple attempts failed to follow the course of the ureter to the renal pelvis. Subsequently the renal pelvis was directly opened through the mesocolon and a flexible cystoscope was used to basket the stone out. Estimated Blood Loss was <100ml. The patient was discharged 2 days postoperatively.

**Conclusion:**

Robotic pyelolithotomy is safe and feasible for management of ectopic pelvic kidneys with pelvic stones. The use of flexible cystoscopy helped in localizing and extracting the stone in our case. Detailed understanding of patient’s anatomy helps in the success of this procedure.

**Available at: http://www.intbrazjurol.com.br/video-section/al-yousef_374_374**

